# In vitro infectivity and differential gene expression of *Leishmania infantum* metacyclic promastigotes: negative selection with peanut agglutinin in culture versus isolation from the stomodeal valve of *Phlebotomus perniciosus*

**DOI:** 10.1186/s12864-016-2672-8

**Published:** 2016-05-20

**Authors:** Pedro J. Alcolea, Ana Alonso, María A. Degayón, Mercedes Moreno-Paz, Maribel Jiménez, Ricardo Molina, Vicente Larraga

**Affiliations:** Laboratorio de Parasitología Molecular, Departamento de Microbiología Molecular y Biología de las Infecciones, Centro de Investigaciones Biológicas (Consejo Superior de Investigaciones Científicas), calle Ramiro de Maeztu, 9, 28040 Madrid, Spain; Laboratorio de Ecología Molecular, Centro de Astrobiología, (Instituto Nacional de Técnica Aeroespacial “Esteban Terradas”-Consejo Superior de Investigaciones Científicas), ctra. de Ajalvir Km 4, 28850 Torrejón de Ardoz, Madrid Spain; Unidad de Entomología Médica, Servicio de Parasitología, Centro Nacional de Microbiología, Virología e Inmunología Sanitarias (Instituto de Salud Carlos III), ctra. Majadahonda-Pozuelo s/n, 28220 Majadahonda, Madrid Spain

**Keywords:** *Leishmania infantum*, Metacyclic promastigotes, *Phlebotomus perniciosus*, Peanut lectin agglutination, Infectivity, Differential gene expression

## Abstract

**Background:**

*Leishmania infantum* is the protozoan parasite responsible for zoonotic visceral leishmaniasis in the Mediterranean basin. A recent outbreak in humans has been reported in this area. The life cycle of the parasite is digenetic. The promastigote stage develops within the gut of phlebotomine sand flies, whereas amastigotes survive and multiply within phagolysosomes of mammalian host phagocytes. The major vector of *L. infantum* in Spain is *Phlebotomus perniciosus*. The axenic culture model of promastigotes is generally used because it is able to mimic the conditions of the natural environment (i.e. the sand fly vector gut). However, infectivity decreases with culture passages and infection of laboratory animals is frequently required. Enrichment of the stationary phase population in highly infective metacyclic promastigotes is achieved by negative selection with peanut agglutinin (PNA), which is possible only in certain *Leishmania* species such as *L. major* and *L. infantum*. In this study, in vitro infectivity and differential gene expression of cultured PNA-negative promastigotes (Pro-PNA^−^) and metacyclic promastigotes isolated from the sand fly anterior thoracic midgut (Pro-Pper) have been compared.

**Results:**

In vitro infectivity is about 30 % higher in terms of rate of infected cells and number of amastigotes per infected cell in Pro-Pper than in Pro-PNA^−^. This finding is in agreement with up-regulation of a leishmanolysin gene (gp63) and genes involved in biosynthesis of glycosylinositolphospholipids (GIPL), lipophosphoglycan (LPG) and proteophosphoglycan (PPG) in Pro-Pper. In addition, differences between Pro-Pper and Pro-PNA^−^ in genes involved in important cellular processes (e.g. signaling and regulation of gene expression) have been found.

**Conclusions:**

Pro-Pper are significantly more infective than peanut lectin non-agglutinating ones. Therefore, negative selection with PNA is an appropriate method for isolating metacyclic promastigotes in stationary phase of axenic culture but it does not allow reaching the in vitro infectivity levels of Pro-Pper. Indeed, GIPL, LPG and PPG biosynthetic genes together with a gp63 gene are up-regulated in Pro-Pper and interestingly, the correlation coefficient between both transcriptomes in terms of transcript abundance is *R*^2^ = 0.68. This means that the correlation is sufficiently high to consider that both samples are physiologically comparable (i.e. the experiment was correctly designed and performed) and sufficiently low to conclude that important differences in transcript abundance have been found. Therefore, the implications of axenic culture should be evaluated case-by-case in each experimental design even when the stationary phase population in culture is enriched in metacyclic promastigotes by negative selection with PNA.

**Electronic supplementary material:**

The online version of this article (doi:10.1186/s12864-016-2672-8) contains supplementary material, which is available to authorized users.

## Background

Leishmaniasis is a neglected vector-borne parasitic disease caused by protozoan parasites grouped into the genus *Leishmania* (Kinetoplastida: Trypanosomatidae). The estimated prevalence is 12 million people worldwide. The most severe clinical manifestation is visceral leishmaniasis (VL), which is fatal without treatment. About 60,000 deaths by VL are declared annually [1, 2]. *L. infantum* is responsible for zoonotic VL in the Mediterranean basin and co-infection with HIV has been reported [3, 4]. Cutaneous and visceral signs are observed in the clinical profile of the canine reservoir. Recently, an outbreak in humans has been reported in central Spain, being hares reservoirs probably [5–7]. The life cycle of the parasite involves two stages: promastigotes and amastigotes. The promastigote is the fusiform motile extracellular stage with a flagellum emerging from the cellular body and the amastigote is the spherical immobile stage with a non-emergent flagellum. The developmental process of promastigotes is known as metacyclogenesis [8]. This process takes place within the gut of hematophagous sand flies (Diptera: Psychodidae, Phlebotominae), where different promastigote stages are observed (procyclics, haptomonads, nectomonads, leptomonads and metacyclics) [9]. When a sand fly vector feeds from a mammalian host, metacyclic promastigotes are injected in the dermis. Of those, few are internalized by phagocytes and differentiated to the amastigote stage under nitrosative stress, acidic pH, increased temperature and the activity of acid hydrolases. *Phlebotomus perniciosus* is the most common sand fly vector in the center and the West of the Mediterranean basin [10, 11].

Several proteins are anchored to the surface of promastigotes through glycosylphosphatidylinositol (GPI). The gp63 surface protein (leishmanolysin) is an important metalloprotease associated to resistance to lysis by the complement system [12]. Other major molecules anchored to the plasma membrane are the lipophosphoglycan (LPG), the membrane-bound proteophosphoglycan (mPPG) and glycosylinositol phospholipids (GIPLs). It has been suggested that GIPLs protect the parasite against the hydrolytic enzymes of the parasitophorous vacuole (reviewed by [13]).

The sand fly gut is the natural microenvironment of promastigote differentiation to more infective non-proliferative metacyclic forms [14–16]. This process is often mimicked in vitro by axenization and culture at 26–27 °C in undefined media containing heat inactivated mammalian serum [17–22]. The main advantage of axenic cultures is that plenty of promastigote biomass is produced. However, attenuation of infectivity and virulence is observed accross culture passages, which is often remedied by passages through laboratory animals (reviewed in [21]). Differences between promastigotes in culture and within the sand fly in terms of promastigote development to the amastigote stage were already reported [23, 24].

Sacks and Perkins [15] described that procyclic *L. major* promastigotes, located in the abdominal gut of the sand fly, were not infective. Conversely, metacyclic promastigotes, located in the anterior part of the thoracic midgut, were able to produce infection in mice. Metacyclogenesis also takes place in axenic culture [16, 25]. Isolation of metacyclic *L. major* and *L. infantum* promastigotes is performed in culture on the basis of differential agglutination properties with the *Arachys hypogaea* lectin, the peanut agglutinin (PNA). Procyclic promastigotes are able to agglutinate because the lectin binds to the galactose residues of the LPG. These residues are blocked by arabinose ones that are added in the ongoing of metacyclogenesis leading to the loss of the agglutination capability. A differential centrifugation procedure allows the isolation of agglutinating procyclic (Pro-PNA^+^) and non-agglutinating metacyclic (Pro-PNA^−^) promastigotes within the stationary phase of axenic culture. Agglutination is reversible because dilution of the suspension leads to disappearance of the agglutination complexes. For this reason, additional PNA is required to maintain the appropriate concentration when the pellet is resuspended during the negative selection procedure of Pro-PNA^−^ [26]. Genes related with infectivity are up-regulated in the minor Pro-PNA^−^ promastigote subpopulation, which is more infective than the Pro-PNA^+^ in *L. infantum* [26], as well as in *L. major* [25, 27–29].

Transcriptome analysis of metacyclic promastigotes isolated from the gut of the sand fly (Pro-Pper) is possible thanks to mRNA amplification [23]. Comparative in vitro infection and high throughput transcriptome analyses of Pro-Pper versus Pro-PNA^−^ has been performed and their infectivity has been compared in vitro. Herein, we confirm that Pro-Pper metacyclic promastigotes are more infective than Pro-PNA^−^.

## Methods

### Ethics statement

Blood samples were extracted from a New Zealand White rabbit to feed *P. perniciosus* sand flies during infection with a suspension of phagocytic cells infected with *L. infantum*. The protocol was performed according to the EU (2010/63) and Spain (RD1201/2005) regulations and it was approved by the ISCIII Ethics Committee for Research in Animal Welfare (license CBA PA73-2011).

### Promastigote axenic culture

Promastigotes of the MCAN/ES/98/10445 isolate (zymodeme MON-1) of *Leishmania infantum* were cultured at 27 °C in RPMI 1640 supplemented with L-glutamine (Lonza-Cambrex, Karlskoga, Sweden), 10 % heat inactivated fetal bovine serum (HIFBS) (Lonza-Cambrex) and 100 μg/ml streptomycin – 100 IU/ml penicillin (Lonza-Cambrex). The inoccula were used at the 5^th^ passage after they had been obtained from the sand fly gut [23].

### Negative selection of metacyclic promastigotes with PNA

Stationary phase promastigotes were harvested at 2,000 g for 10 min and resuspended at a cell density of 2 x 10^8^ cells/ml in 10 ml complete medium containing 50 μg/ml PNA [30]. Promastigotes were allowed to agglutinate at room temperature for 30 min. Then, the sediment and the supernatant were recovered. The former was diluted to the initial volume in fresh complete medium containing 50 μg/ml PNA. Both fractions were centrifuged at 200 g for 10 min and the supernatants obtained were centrifuged at 2,000 g to obtain PNA^−^ promastigotes (Pro-PNA^−^). All steps were checked at the light microscope.

### In vitro infection of the human U937 myeloid cell line

The human cell line U937 (ATCC® CRL1593.2), originally obtained from a patient with histiocytic leukemia [31], was infected in vitro with *L. infantum* promastigotes for two different purposes: infection of sand flies to obtain promastigotes from the stomodeal valve (Pro-Pper) and evaluation of in vitro infectivity of Pro-PNA^−^ and Pro-Pper. First, the U937 cell line was cultured at 37 °C in complete medium in the presence of 5 % CO_2_ for 72 h. Then, the cells were centrifuged at 250 g and differentiated in complete medium by stimulation with 20 ng/ml phorbol 12-myristate 13-acetate (Sigma, Saint Louis, MO) for 72 h [32]. This step was performed in a 175 cm^2^ flask when the resulting infected U937 cells were diluted in rabbit blood to infect sand flies experimentally (see below). In the case of evaluation of the in vitro infection capability of promastigotes, infections of U937 cells were performed over 8-well cell chamber slides (LabTek, New York, NY). The cultures were mildly rinsed with RPMI supplemented with L-glutamine (Lonza-Cambrex). Only, cells cultured in flasks were detached by vigorous shaking in the presence of 0.5 g/l trypsin, 0.2 g/l EDTA (Lonza-Cambrex). Trypsin was inactivated by adding one volume of complete medium. The differentiated cells were recovered by centrifugation. Then, they were mixed with stationary phase promastigotes at a promastigote:cell ratio 20:1 and incubated at 37 °C in complete medium in a water bath for 2 h. The mixture was mildly mixed every 15 min. Once this incubation step was over, the cells were harvested and incubated again in the culture flasks in complete medium at 37 °C, in an atmosphere of 5 % CO_2_ for 72 h. The cultures were rinsed with complete medium after 2 h and 16 h. Infections were checked at the light microscope with Giemsa stain prior to sand fly feeding. In the case of differentiated cells attached to the 8-well chamber slides, infections were performed at 37 °C at a promastigote:cell ratio 5:1 in 400 μl complete medium in an atmosphere of 5 % CO_2_ for 2 h. Next, the cells were rinsed with complete medium at 2 and 16 h post-infection as in the previous procedure. The incubations were resumed and samples were taken at 24, 48 and 96 h post-infection to estimate the percentage of infected cells and the number of amastigotes per infected cell (100 cells were counted per sample). For this purpose, three more washes were performed prior to treatment with hypotonic solution (180 μl complete medium diluted with 220 μl water per well) for 5 min. Four washes with 150 μl ethanol-acetic acid 3:1 were carried out once the hypotonic solution had been removed. Then, fixation was performed with the same solution for 10 min and this step was repeated three times. Finally, cells were allowed to air dry and the wells removed from the slide. Modified Giemsa staining was performed with Diff-Quick® Stain Solution I and II (Dade Behring, Marburg, Germany). The preparations were washed with distilled water, air dried and mounted with Entellan® Neu (Merck, Darmstadt, Germany). The percentage of infected cells and the number of amastigotes per infected cell were estimated in three biological replicate experiments and the statistical analysis was based on the Student’s paired t-test.

### Infection of *P. perniciosus* and isolation of metacyclic promastigotes from the stomodeal valve

**Fig. 1:**
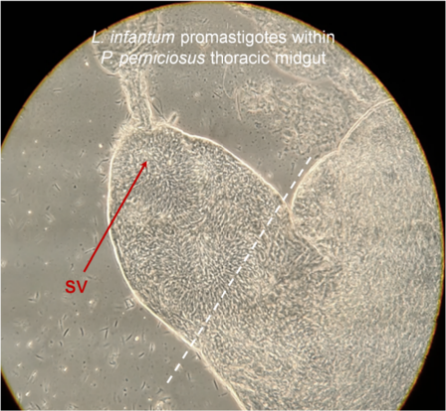
Isolation of Pro-Pper. Promastigotes within the stomodeal valve of *P. perniciosus*. Sand flies were dissected and the guts separated. The abdominal gut and the posterior part of the thoracic midgut were discarded and Pro-Pper were recovered from the anterior part of the thoracic midgut (discontinuous line). The Pro-Pper population is enriched in metacyclic promastigotes as they are near the stomodeal valve (SV). Carryover of leptomonads was minimized by recovering just promastigotes in suspension and avoiding gut tissue as much as possible. However, it is assumed that this population is heterogeneous as expected in any biological experiment, as well as the Pro-PNA^−^ population

An established colony of *P. perniciosus* sand flies [33] was maintained in a climatic chamber at 27–28 °C, 90–100 % relative humidity, 17 h light - 7 h darkness photoperiod in the presence of a 30 % fructose solution. About 150–200 sand flies were fed with a suspension of 2 x 10^6^ infected U937 cells in 2 ml of defibrinated rabbit blood over a chicken skin membrane [34]. Sand fly samples were dissected daily in order to follow the course of infection. After 5 days, sand flies were dissected daily with the additional purpose of extracting the guts for isolation of mature promastigotes from the anterior thoracic midgut close to the stomodeal valve (Fig. 1) and subsequent preparation of RNA samples (see below). For this purpose, the gut was dissected to isolate the anterior part of the thoracic midgut and recover promastigotes in PBS with a Pasteur pipette. Three independent samples were prepared. Each one included promastigotes from about 20 infected sand flies. The replicate samples finally used had been obtained the day before the death phase started (day 6). This was also applicable for the equivalent Pro-PNA^−^ population (see above).

### RNA isolation, mRNA amplification and synthesis of labeled cDNA

Total RNA was purified from Pro-Pper and Pro-PNA^−^ with TRizol® reagent (Life Technologies, Carlsbad, CA) according to the manufacturer’s instructions. One μg per ml of glycogen (Life Technologies) was added as carrier to the aqueous phase just before isopropanol precipitation. mRNA was doubly amplified (aaRNA) using MessageAmp^TM^ II aRNA Amplification Kit (Life Technologies) as previously described [24]. RNA quality was assessed with the Agilent 2100 Bioanalyzer (Life Technologies) in an RNA 6000 NanoChip according to the manufacturer's instructions.

The first strand aminoallyl-cDNA was synthesized using 10 μg aaRNA template. First, aaRNA was mixed with 6 μg of random hexamer primers (Life Technologies). The mixture was denatured at 70 °C for 10 min and immediately cooled. Then, first strand synthesis was performed at 46 °C in 30 μl final reaction volume for 3 h with 570 μM dATP, dCTP and dGTP, 230 μM dTTP, 340 μM aminoallyl-dUTP, 10 μM DTT and 600 U SuperScript® Reverse Transcriptase (Life Technologies). Next, RNA was degraded at 70 °C in 100 mM NaOH/10 mM EDTA for 30 min and the solution was then neutralized with 3 μl of 3 M sodium acetate pH5.2. Aminoallyl-cDNA was purified with QiaQuick PCR Purification Kit (Qiagen, Hilden, Germany) according to the manufacturer’s instructions except for two buffers: custom phosphate wash (5 mM KPO_4_, 80 % ethanol, pH8.0) and phosphate elution (4 mM KPO_4_) buffers replaced those provided in the kit to avoid blockage of the amino group. Purified aminoallyl-cDNA was completely dried in a vacuum centrifuge and resuspended in 10 μl of water. A solution containing the cyanine monofunctional dyes (Cy3 and Cy5; GE Healthcare, Chalfont Saint Giles, UK) were prepared at 12 ng/μl in DMSO. Coupling was allowed at room temperature in darkness for 1 h once 5 μl of the Cy3 or Cy5 solution was added to the respective samples (Cy3 for Pro-PNA^−^ and Cy5 for Pro-Pper). Finally, labelled cDNA was purified with QiaQuick PCR Purification Kit (Qiagen) according to the manufacturer’s instructions.

### Microarray hybridization analysis of differential gene expression

*L. infantum* shotgun genome microarrays [26] were washed in 0.1 % N-lauroylsarcosine in 2X SSC, then in 2X SSC. The slides were heated at 95 °C for 3 min and immediately chilled in 100 % ethanol (10 s after removal from boiling water) thus denaturing and fixing DNA. The slide was spin dried in a minicentrifuge and attached upside down over a Hybri-Slip coverslip (Sigma) containing a 60 μl drop of 3X SSC, 0.3 % N-lauroylsarcosine, 60 mMTris-HCl pH8.0, 83 ng/ml denatured herring sperm DNA and 1 % BSA. Blocking was allowed at 42 °C for 30 min using a hybridization chamber submerged in a water bath. Then, blocked microarrays were incubated at 40 °C for 16 h with a mixture of the Cy3- and Cy5-labelled cDNA samples (50 pmol dye each) in hybridization solution (equal to blocking solution except for 0.1 % BSA, 25 ng/ml poly(T) and 50 % deionized formamide). The slide was washed three times, first in 2X SSC, 0.2 % SDS at 40 °C, then in 1X SSC at room temperature and finally in 0.2X SSC at room temperature.

Hybridized microarrays were scanned with GenePix 4100A (Axon, Foster City, CA). Local feature background was subtracted from raw fluorescence intensity values with GenePix Pro 7.0 software. Raw data were normalized by the LOWESS per pin algorithm and Student’s t-test contrast considering three biological replicates was performed with AlmaZen software (BioAlma, Tres Cantos, Spain). Differential expression cutoff values were applied to obtain the set of clones containing differentially regulated genes: (i) fold change F ≥ 2 (Cy5/Cy3 ratio if Cy5 > Cy3) or F ≤ −2 (−Cy3/Cy5 ratio if Cy3 > Cy5), (ii) total relative fluorescence intensity value > 5000 arbitrary fluorescence units and (iii) p* < 0.05. The clones selected on the basis of these conditions were grown and sequenced with the M13-pUC18 primers and assembled as described [26]. These clones were classified according to the following genome alignment outcomes: (i) e-value < 1e-10 for both ends, (ii) convergent orientation in the genome sequence and (iii) clone length ≤ 11 kbp [26]. Type *a* clones were defined by a unique pair of alignments. Type *b* clones presented more than a pair of alignments due to adjoining sequence repeats; the best sequence identity is considered in this case. Finally, *c* clones did not completely fulfill all three requirements, which is mostly due to the presence of two or more inserts in the clone or the lack of one of the end sequences. Clones were then associated to annotated genes using a Perl script.

### Real time quantitative RT-PCR (qRT-PCR)

Synthesis of unlabeled single stranded cDNA was performed as indicated above except for the dNTP mixture (10 mM each dATP, dCTP, dGTP and dTTP in this case) The design of primers and FAM-MGB probes (Additional file 1: Table S1), configuration of 384-well plates and in situ synthesis was managed by Custom TaqMan® Assays-by-Design (Life Technologies). The qRT-PCR assays were run in a 7900HT Fast Real Time PCR system (Life Technologies) once cDNA templates and TaqMan® Universal Master Mix (Life Technologies) were added. Three sample replicates and three 1/10 dilutions of each one were included (25, 2.5 and 0.25 ng cDNA in 15 μl final reaction volume). Thermal cycling conditions were: 95 °C for 5 min; 40 x [95 °C for 30s; 60 °C for 1 min, data acquisition]. A 20 % coefficient of variation cutoff was applied and PCR efficiencies were calculated by the standard curve best fit method [35]. Normalized quantities (Q_n_) were calculated by dividing the efficiency-corrected raw quantity values (equal to efficiency to the power of –Ct) of the gene of interest by those of the reference gene (*L. infantum* gGAPDH). Then, F was obtained by dividing Q_n_ of both experimental conditions (Pro-Pper/Pro-PNA^−^) for each dilution. The mean F value and the SD were calculated considering Q_n_ values of all dilutions.

## Results and discussion

### Isolation of Pro-Pper and evaluation of in vitro infection

In 1985, it was described that *L. major* PNA^−^ promastigotes are more infective than PNA^+^ [27]. This was corroborated in *L. infantum* by us and up-regulated genes involved in infectivity were found [26]. In this study, Pro-Pper and Pro-PNA^−^ have been compared in terms of in vitro infectivity and differential gene expression.

**Fig. 2:**
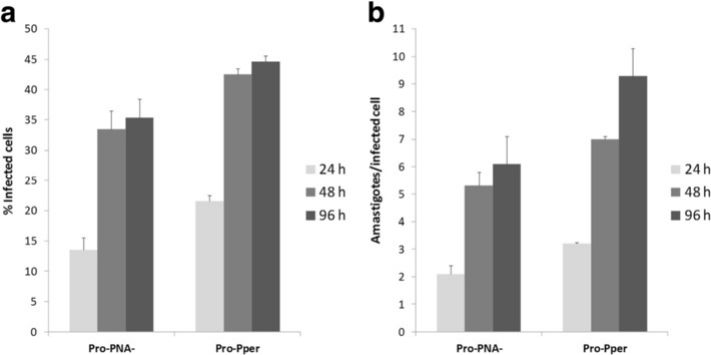
In vitro infection of the stimulated U937 cell line with Pro-PNA^−^ and Pro-Pper. All differences are statistically significant (unpaired Student's t-test, *p* < 0.001). **a** Infection rate (%). Mean ± SD (Pro-PNA^−^and Pro-Pper, respectively): 13 ± 2 and 21 ± 1 (24 h); 33 ± 3 and 42 ± 1 (48 h); 35 ± 3 and 45 ± 1 (96 h). **b** Number of amastigotes per infected cell. Mean ± SD (Pro-PNA^−^ and Pro-Pper, respectively): 2.1 ± 0.3 and 3.2 ± 0.0 (24 h); 5.3 ± 0.5 and 7.0 ± 0.1 (48 h); 6.0 ± 1 and 9 ± 1 (96 h)

Digestive tracts were obtained from sand flies once they were dissected. The anterior thoracic midgut was selected (Fig. 1). Then, Pro-Pper promastigotes in suspension were recovered and carryover of gut tissue (and therefore leptomonads) was minimized. Three biological replicates of the experiments were performed. Pro-Pper samples obtained for evaluation of in vitro infection of U937 cells were recovered in PBS and immediately resuspended in 200 μl complete medium. The suspension was added to PMA-differentiated U937 cells attached to 8-well slides and allowed to infect. After 2 h at 37 °C, 5 % CO_2_, the culture was washed to eliminate remaining promastigotes. Finally, samples were obtained at 24, 48 and 96 h post-infection. The same procedure was followed for Pro-PNA^−^. Then, the percentage of infected cells and the number of amastigotes per infected cell were estimated. Statistical analysis was performed by the unpaired Student’s t test. The reduction of in vitro infectivity in Pro-PNA^−^ with respect to Pro-Pper in terms of rate of infected cells is 38, 22 and 22 % at 24, 48 and 96 h post-infection, respectively (Fig. 2a). The reduction in terms of average number of amastigotes per infected cell is 35, 25 and 33 % at 24, 48 and 96 h post-infection, respectively (Fig. 2b). The differences were statistically significant in all cases (*p* < 0.001). Therefore, culture reduces the infection ability of promastigotes even when metacyclics are obtained by negative selection with PNA. Hence, this procedure is an appropriate method for isolation of metacyclics in culture but worse than isolation from the natural environment, i.e. the sand fly anterior thoracic midgut (Fig. 2).

### Transcript amplification and microarray hybridization analysis

Pro-Pper samples used for gene expression analysis were immediately washed once in PBS and resuspended in TRIzol® reagent (Life Technologies). Then, two rounds of mRNA amplification were performed to obtain enough material for the high-throughput gene expression analysis. Obviously, Pro-PNA^−^ sample processing was the same.

**Fig. 3:**
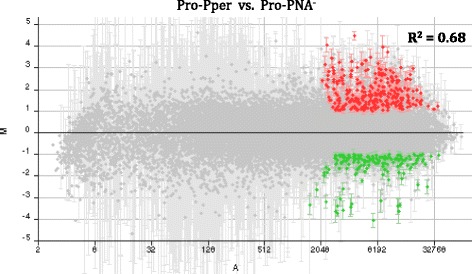
M/A scatter plot of the three-replicate Pro-Pper/Pro-PNA^−^ microarray hybridization experiment. M = (log_2_Ri – log_2_Gi) and A = [(log_2_Ri + log_2_Gi)/2], where R and G are, respectively, red (Cy5) and green (Cy3) fluorescence intensity values. Red spots represent selected clones that contain a gene up-regulated by at least 2-fold and green spots represent those down-regulated by at least 2-fold. The Pearson correlation coefficient (R^2^) is provided

Several genes had been included in the microarrays as positive hybridization controls [26]. The stage-specific A2 amastigote gene [36] is not differentially expressed between Pro-Pper and Pro-PNA^−^ (Additional file 2: Table S2) as expected. The fluorescence intensity values (FI) of all negative microarray hybridization controls are below the average background level as expected (Additional file 2: Table S2). The origin of these genes is the extremophile *Leptospirillum ferrooxidans* [26]. In total, 174 differentially regulated genes have been found: 111 are up-regulated in Pro-Pper and 63 in Pro-PNA^−^ (Fig. 3, Tables 1 and 2, Additional file 3: Tables S3-S5). The Pearson correlation coefficient between Pro-PNA^−^ and Pro-Pper in terms of normalized fluorescence intensity values is *R*^2^ = 0.68 (Fig. 3).

**Table 1:** Absolute frequencies of differentially regulated genes in Pro-Pper/Pro-PNA^−^

Annotation status	Frequency of differentially regulated genes in Pro-Pper/Pro-PNA^−^	
	Up-regulated	Down-regulated
Genes of known function	53	26
Hypothetical protein genes	56	31
Type c clones	2	6
Total (*n* = 174)	111	63

**Table 2:**
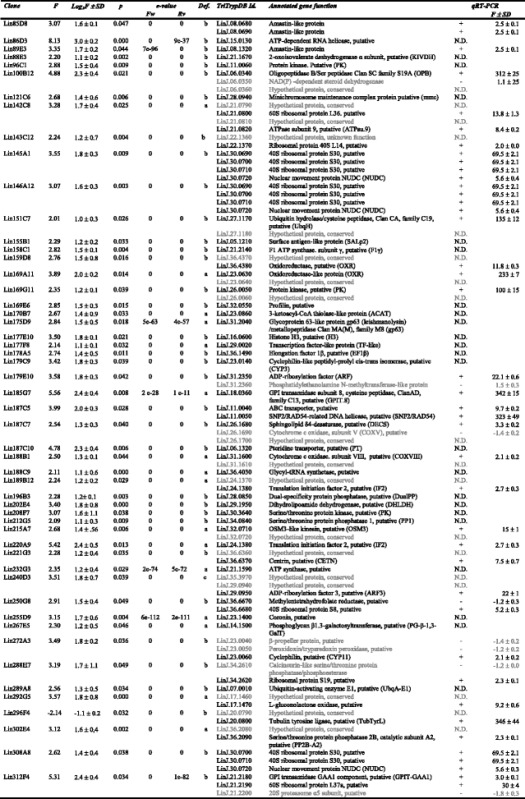
Genes of known function up-regulated in Pro-Pper/Pro-PNA^−^

Features described: clone number, fold change (up-regulation if *F* ≥ 2.0), log_2_F and standard deviation (SD), Student's t-test *p*-value (p), clone definition (Def.; see Methods), TriTrypDB identifier, annotated gene function (including abbreviations defined in the text) and qRT-PCR outcome. Genes in grey (clones that overlap with more than one annotated gene): they are not differentially regulated (confirmed by qRT-PCR) or there is no evidence to support that they are differentially regulated in other cases (not determined by qRT-PCR)

### qRT-PCR analysis

Most clones overlapping with more than one gene annotation were analyzed by the TaqMan Probe qRT-PCR approach. Therefore, large clones that represent more than one CDS could be resolved. This approach was also useful to validate 26.3 % of the microarray results (Tables 2 and 3), together with the internal hybridization controls already mentioned (Additional file 2: Table S2). Constant expression values were obtained just in the case of certain clones overlapping with more than one gene.

**Table 3:**
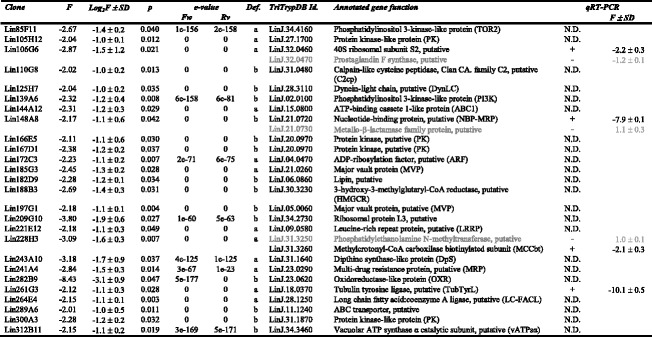
Genes of known function down-regulated in Pro-Pper/Pro-PNA^−^

Features described: clone number, fold change (down-regulation if *F* ≤ −2.0), log_2_F and standard deviation (SD), Student's t-test *p*-value (p), clone definition (Def.; see Methods), TriTrypDB identifier, annotated gene function (including abbreviations defined in the text) and qRT-PCR outcome. Genes in grey (clones that overlap with more than one annotated gene): they are not differentially regulated (confirmed by qRT-PCR) or there is no evidence to support that they are differentially regulated in other cases (not determined by qRT-PCR)

### Differential gene expression between Pro-Pper and Pro-PNA^−^

In a study of differentiation of promastigotes to amastigotes, Lahav et al. [37] described that relative transcript levels do not correspond with abundance of the encoded protein in many cases. Indeed, coincidence was observed in just about 25 % cases in quantitative terms (Pearson correlation coefficient). However, qualitative coincidences (constitutive expression, up-regulation and down-regulation) were observed in 65 % cases (589 out of 902 genes). As we have seen, one of the purposes of this study is comparing Pro-PNA^−^ and Pro-Pper in terms of relative transcript abundance, focusing on differentially regulated genes. For this purpose, the qualitative information is more relevant than the quantitative because it provides a picture of the steady-state transcript levels in both conditions. Hence, insight into the adequacy of negative selection of metacyclics in culture with PNA compared to the natural environment (the sand fly gut) is provided herein. Additionally, the different approaches used (e.g. microarray hybridization analysis and qRT-PCR in this study) have different dynamic ranges and sometimes provide different quantitative results for coincident qualitative results. In conclusion, the transcriptome analysis is useful in this study and it is the only possibility to study gene expression in Pro-Pper so far. In fact, the negligible amount of parasite material obtained from sand fly dissections does not allow performing analysis of individual proteins and the proteome, whereas it is possible to amplify RNA. In our case, 174 genes are differentially regulated between Pro-PNA^−^ and Pro-Pper. Therefore, we expect about 113 genes showing the same qualitative outcome of relative abundance at the transcript and protein levels.

**Fig. 4:**
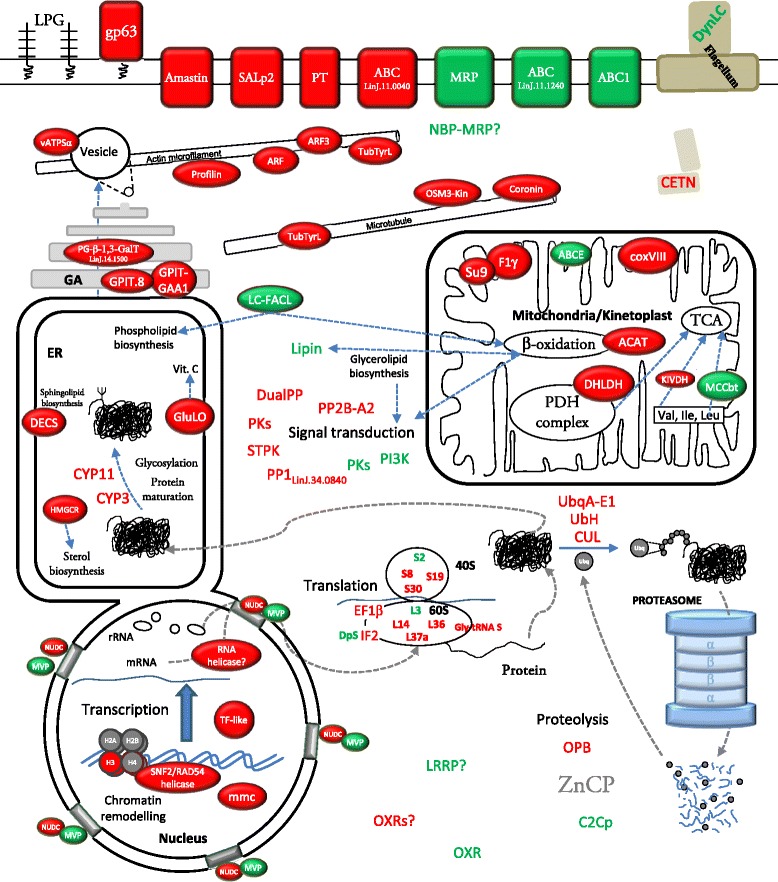
Differentially regulated genes of known function in Pro-Pper/Pro-PNA^−^. Protein products in red correspond to genes up-regulated in Pro-Pper and those in green to Pro-PNA^−^. See abbreviations in the text and in Tables 2 and 3

**Fig. 5:**
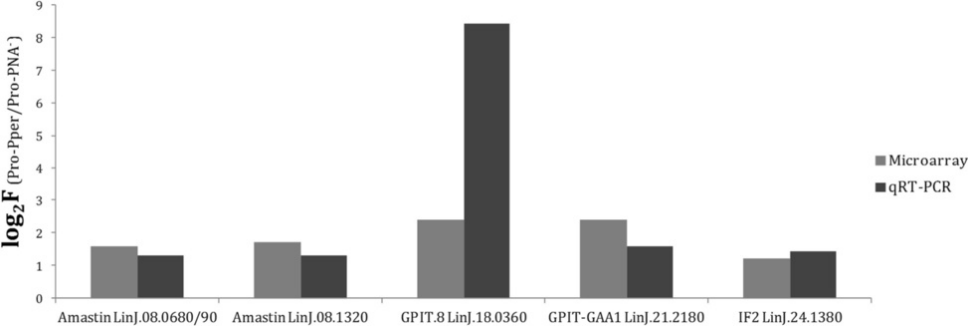
Up-regulation of genes encoding amastins, GPI biosynthetic proteins and the IF2 in Pro-Pper. The results of the qRT-PCR and microarray hybridization analyses (Table 2) are compared

The comprehensive study of the differences found in transcript abundance is illustrated in Fig. 4. The most striking qPCR results are illustrated in Fig. 5. Provided that unequivocal identification is very important [38], gene name abbreviations have been included in Tables 2 and 3. Unless otherwise indicated, the term up-regulation refers to Pro-Pper and down-regulation to Pro-PNA^−^.

#### Chromatin structure, nucleocytoplasmic transport and regulation of gene expression at the post-transcriptional and post-translational levels

Three up-regulated genes in Pro-Pper involved in DNA replication and chromatin remodeling have been found: histone (H3), SNF2/RAD54 helicase and minichromosome maintenance protein (mcm). Metacyclic promastigotes are non-dividing forms of the parasite, but H3 gene over-expression is in agreement with independence of mRNA levels from DNA synthesis [39].

A nuclear movement protein gene (NUDC) is up-regulated in Pro-Pper, whereas two major vault protein genes (MVP) are down-regulated. This suggests differences in nucleocytoplasmic transport and signaling between Pro-Pper and Pro-PNA^−^. In fact, MVPs are the main constituents of vaults, which are protein complexes that may participate in both processes [40] and they are able to interact with the target of rapamycin protein 4 (TOR4) in *Trypanosoma brucei* [41].

A transcription factor-like protein gene (TF-like) and the RNA helicase LinJ.15.0130 are up-regulated in Pro-Pper. As for translation regulation, several genes are differentially regulated between both populations of metacyclics. On the one hand, the elongation factor 1β (EF1β), the initiation factor 2 (IF2), the 40S ribosomal proteins S8, S19 and S30, the 60S ribosomal proteins L14, L36 and L37a and the glycyl-tRNA synthetase are up-regulated. On the other hand, the diphthine synthase gene, the ribosomal proteins S2 and L3 are down-regulated. The EF1β is up-regulated by cadmium [42], whereas the IF2 is up-regulated in stationary phase promastigotes with respect to intracellular amastigotes [24]. Diphthine is the direct precursor of diphthamine, a molecule able to inactivate the IF2 by addition of a residue of ADP-ribose, as the diphtheria toxin does [43]. Down-regulation of the diphthine synthase (DpS) in Pro-Pper (Table 3) is in agreement with up-regulation of the IF2. Finally, the cyclophilin genes CYP3 and CYP11, involved in protein folding, are up-regulated.

#### Proteolysis

The oligopeptidase B gene (OPB) is up-regulated in Pro-Pper, whereas the calpain-like cysteine peptidase (C2Cp) LinJ.31.0480 is down-regulated. The results found also suggest changes in ubiquitin-proteasome protein degradation pathway between Pro-Pper and Pro-PNA^−^, as the ubiquitin activating enzyme E1 gene (UbqA-E1), the E3 activating protein cullin and the ubiquitin hydrolase (UbH) are up-regulated.

#### Protein-protein interaction

A leucine-reach repeat protein (LRRP) of unknown function in the parasite is up-regulated in Pro-PNA^−^. LRRPs have been associated to functions generally involving protein-protein interactions in other organisms such RNase inhibitors, tropomyosin, tropomodulin and toll-like receptors. Each LRR motif has a sheet-turn-helix structure [44].

#### Metabolism, transport and signal transduction

The dihydrolipoamide dehydrogenase gene (DHLDH) is up-regulated in Pro-Pper, as well as some genes that participate in the respiratory chain. Namely, the cytochrome oxidase VIII subunit (coxVIII), the ATPase subunit 9 (ATPsu.9) and the F1γ subunit of the ATP synthetase (F1γ).

The up-regulation of the α-ketoisovalerate dehydrogenase gene (KIVDH) and the down-regulation of the methylcrotonyl-CoA carboxylase biotinylated subunit gene (MCCbt) suggest that isoleucine and valine catabolism may be favored in Pro-Pper, in agreement with the EC 1.2.4.4 and 6.4.1.4 activities of the KIVDH and MCCbt (Tables 2 and 3; TriTrypDB) within the KEGG pathway lif00280 [45].

A thiolase I gene (ACAT) is up-regulated in Pro-Pper, thus suggesting that β-oxidation of fatty acids is more active in this population than in Pro-PNA^−^. On the opposite, the long chain fatty acid:CoA ligase gene (LC-FACL) is down-regulated, which suggests that long chain fatty acids are a more common source for Pro-PNA^−^ to feed the β-oxidation degradation pathway or to contribute to fatty acid, glycerolipid and phospholipid biosynthesis (EC 6.2.1.3. activity in KEGG pathways ec00071 and ec00061, respectively). The lipin gene is also down-regulated and it is involved in glycerolipid biosynthesis. Consequently, these data suggest that β-oxidation is more active in Pro-Pper, whereas the lipid biosynthetic processes would be favored in Pro-PNA^−^. Both pathways provide molecules that are active in signaling processes [46]. The sphingolipid biosynthetic pathway may be more active in Pro-Pper as suggested by up-regulation of the sphingolipid Δ^4^-desaturase gene (DECS). Sphingolipids are also able to develop signaling functions in the parasite [47].

Protein kinases of these parasites have been identified [48] but most signaling pathways are still not known in these organisms yet [49]. The genes encoding a serine/threonine protein phosphatase 1 LinJ.34.0840 (PP1), a serine/threonine protein phosphatase 2 catalytic subunit A2 (PP2B-A2), a dual specificity protein phosphatase (DualPP) and three PKs are up-regulated, whereas the phosphatidylinositol 3-kinase gene (PI3K) and a protein kinase gene (PK) are down-regulated.

The genes encoding the α subunit of the vacuolar ATP synthetase (vATPSα) and the ABC transporters ABCE and ABC LinJ.11.0040 are up-regulated in Pro-Pper, whereas the multidrug resistance protein (MRP), the ABC LinJ.11.1240 and the ABC1 are down-regulated. Finally, a nucleotide binding protein (NBP-MRP), probably an MRP (see LinJ.21.0720 entry in TriTrypDB), is also down-regulated.

#### Cytoskeleton

Several genes encoding actin- and tubulin-interacting proteins (AIP and TIP) are up-regulated in Pro-Pper. The AIPs are the profilin, two ADP-ribosylation factors (ARF LinJ.31.2350 and ARF3) and the tubulin-tyrosine ligase (TubTyrL). The TubTyrL is also a TIP, as well as a coronin and the OSM3-like kinesin. A different ARF gene (LinJ.04.0470) is down-regulated. The ARF1 has been characterized in *T. cruzi* and in *L. donovani,* where it is involved in coatomer assembly in budding vessicles in the secretory pathway and endocytosis [50, 51]. The profilin may be involved in the actin microfilament polymerization machinery [52]. Coronins of *Leishmania* spp., *Trypanosoma* spp. and other protozoan parasites are involved in proliferation, locomotion and phagocytosis [53].

#### Surface molecules

Biosynthesis of glycosylphosphatidylinositol (GPI) may be increased in Pro-Pper, as the genes encoding the GPI transamidase GAA1 component (GPIT-GAA1) and the GPI transamidase subunit 8 (GPIT.8) are up-regulated. The GPI is an essential anchor of important surface molecules, such as the gp63 metalloprotease. One of the genes encoding a gp63 is up-regulated in Pro-Pper. The gp63 has been traditionally associated to metacyclic promastigotes and increased infectivity [12, 54–56]. The GPI is also the essential anchor for glycosylinositolphospholipids (GIPLs) and other surface proteins of the amastigote glycocalix. GIPLs act as receptors for the host cell and as a shield for resistance against lysosomal hydrolases [57]. The GPI anchor is essential for the biosynthesis of GIPLs, which may partially explain the importance of up-regulating GPI-biosynthetic enzymes in metacyclic promastigotes, according to the pre-adaptation hypothesis [24, 58, 59]. The phosphoglycan β-1,3-galactosyltransferase (PG β1,3GalT), also up-regulated in Pro-Pper, is involved in the biosynthesis of the lipophosphoglycan (LPG) and proteophosphoglycans (PPG), which are major surface molecules of promastigotes. The LPG is modified during promastigote differentiation, which makes possible negative selection with PNA.

The amastin superfamily genes LinJ.08.0680/0690/1320 are up-regulated in Pro-Pper vs. Pro-PNA^−^. They were described to be down-regulated in logarithmic phase promastigotes with respect to stationary phase promastigotes [24]. Some of these genes are up-regulated when temperature is raised and pH decreased both in axenic and intracellular amastigotes [24, 60]. Initially, these molecules were thought to be specific of the amastigote stage, but over-expression was also detected in stationary phase promastigotes and metacyclic promastigotes. Hence, they are up-regulated in advance prior to the differentiation process of promastigotes to amastigotes, according to the pre-adaptation hypothesis [24, 58, 59].

### Genes related with infectivity and preparation in advance to life in the phagolysosome

A Zn carboxypeptidase gene from the family M14 (ZnCP) is up-regulated in Pro-PNA^−^ with respect to Pro-PNA^+^ [26]. Despite it is not differentially regulated between Pro-Pper and Pro-PNA^−^, we found that it is up-regulated in Pro-Pper with respect to the whole stationary phase population [61]. This finding together with the differences in infectivity (Fig. 2) support that the degree of differentiation of the Pro-PNA^−^ subpopulation is higher than the whole population in stationary phase as previously reported [26] but lower than Pro-Pper (Fig. 2). The significantly higher infectivity of Pro-Pper promastigotes in terms of rate of infected cells and number of amastigotes per infected cell are in agreement with the higher expression levels of the gp63 gene and GPI, LPG and PPG biosynthetic genes GPIT.8, GPIT-GAA1, PG-β-1,3-GalT. Up-regulation of the SALp2 and amastin genes in Pro-Pper is also probably related. The Pearson correlation coefficient between Pro-Pper and Pro-PNA^−^ in terms of differential gene expression (normalized fluorescence intensity values) is *R*^2^ = 0.68. The meaning of this finding is that both populations are strongly correlated. However, it reveals important differences at the same time because it is not close to the maximum value (1). This is clearly appreciated in the shape of the M/A scatter plot (Fig. 3), which is a non-dispersed (rank −4 < M < 4) dot-cloud simmetric about the M = 0 line (i.e., lack of differential expression).

The gp63 gene, the GPI, LPG and PPG biosynthetic genes and others may be involved in preparation in advance for differentiation and survival of the amastigote stage in the phagolysosome according to the pre-adaptative hypothesis [24, 58, 59]. This is especially probable in the case of the amastin and the GPI biosynthetic protein genes.

## Conclusions

The mean of amastigote counts per infected cell is significantly higher in Pro-Pper than in Pro-PNA^−^, as well as the rate of infected cells. Up-regulation of genes involved in GPI, LPG and PPG biosynthesis and a gp63 gene at the transcript level in Pro-Pper supports the differences found in infectivity. Consequently, the Pro-Pper population is more infective than the Pro-PNA^−^ one. Therefore, Pro-PNA^−^ are not as infective as Pro-Pper, but they are highly infective in any case. This means that enrichment in metacyclics by negative selection with PNA in culture is a good approach but not as good as isolation from the natural environment, i.e. the anterior thoracic midgut of the sand fly. Indeed, the Pearson correlation coefficient (*R*^2^ = 0.68) between both transcriptomes in terms of transcript abundance supports that the similarity between both populations is moderate and the important differences found are presumably related to increased infectivity in Pro-Pper. In other words, the correlation is sufficiently high to consider that both samples are physiologically comparable (i.e. the experiment was correctly designed and performed) and sufficiently low to conclude that important differences in transcript abundance have been found (including genes involved in chromatin structure, nucleocytoplasmic transport, gene expression regulation, signaling and other processes). Therefore, the implications of axenic culture should be evaluated case-by-case in each experimental design even when the stationary phase population is enriched in metacyclic promastigotes by negative selection with PNA.

## Additional files

Additional file 1: Primers and TaqMan-MGB probes used for qRT-PCR validation and determination of differential expression in clones that represent more than one gene. **Table S1.** Sequences of qRT-PCR primers and probes. (XLS 33 kb) Additional file 2: Microarray controls. **Table S2.** The results of the Pro-Per/Pro-PNA^−^ cDNA:genomic-DNA-microarray hybridization analysis for positive and negative control spots. (DOC 48 kb) Additional file 3: Hypothetical proteins. **Table S3.** Hypothetical protein genes up-regulated in Pro-Pper/Pro-PNA^−^. **Table S4.** Hypothetical protein genes down-regulated in Pro-Pper/Pro-PNA^−^. **Table S5.** Type c and qPCR non-determined clones. (DOC 255 kb)

## Abbreviations

aaRNA: doubly amplified mRNA
ABC: ATP-binding cassette
ACAT: thiolase I
AIP: actin-interacting protein
ARF: ADP-ribosylation factor
aRNA: amplified mRNA
ATPsu.9: ATPase subunit 9
C2cp: calpain-like cysteine peptidase, Clan CA, family C2
CETN: centrin
coxVIII: cytochrome oxidase subunit VIII
CYP: cyclophilin
DECS: sphingolipid Δ^4^-desaturase
DHLDH: dihydrolipoamide dehydrogenase
DpS: diphthine synthase
DualPP: dual specificity protein phosphatase
DynLC: dynein light chain
EF1β: elongation factor 1β
GIPL: glycosylinositolphospholipid
gp63: 63 KDa glycoprotein, metalloprotease (leishmanolysin)
GPI: glycosylphosphatidylinositol
GPIT.8: GPI transamidase subunit 8
GPIT-GAA1: GPI transamidase GAA1 component
HIFBS: heat-inactivated fetal bovine serum
HMGCR: 3-hydroxymethylglutaryl-CoA reductase
IF2: initiation factor 2
KIVDH: 2-ketoisovalerate dehydrogenase
LC-FACL: long chain fatty acid:CoA ligase
LPG: lipophosphoglycan
LRRP: leucine-reach repeat protein
MCCbt: methylcrotonyl-CoA carboxylase biotinylated subunit
mPPG: membrane-bound proteophosphoglycan
MRP: multidrug resistance protein
MVP: major vault protein
NBP: nucleotide-binding protein
NUDC: nuclear movement protein
OPB: oligopeptidase B
OXR: oxidoreductase
PDH: pyruvate dehydrogenase complex
PG β1,3GalT: phosphoglycan β-1,3-galactosyltransferase
PI3K: phosphatidylinositol 3-kinase
PK: protein kinase
PNA: peanut agglutinin
PP1: Ser/Thr protein phosphatase 1
PP2B-A2: Ser/Thr protein phosphatase 2, catalytic subunit A2
Pro-PNA^−^: PNA non-agglutinating promastigotes
Pro-Pper: promastigotes isolated from the stomodeal valve of *P. perniciosus*
SALp2: surface antigen-like protein 2
SL-RNA: spliced leader RNA
sPPG: secreted proteophosphoglycan
TF: transcription factor
TIP: tubulin-interacting protein
TOR: target of rapamycin
TubTyrL: tubulin tyrosine ligase
UbH: ubiquitin hydrolase
UbqA-E1: ubiquitin-activating enzyme E1
vATPsα: vacuolar ATP synthetase, subunit α
VL: visceral leishmaniasis
ZnCP: zinc carboxypeptidase, family M14
