# Supplementary material for: In vitro infectivity and differential gene expression of Leishmania infantum metacyclic promastigotes: negative selection with peanut agglutinin in culture versus isolation from the stomodeal valve of Phlebotomus perniciosus
Source: BMC Genomics. 2016 May 20;17:375. doi: 10.1186/s12864-016-2672-8 (PMC4874012; doi:10.1186/s12864-016-2672-8)
Supplement: Additional file 2: — Microarray controls. Table S2. The results of the Pro-Per/Pro-PNA− cDNA:genomic-DNA-microarray hybridization analysis for positive and negative control spots. (DOC 48 kb) [file 12864_2016_2672_MOESM2_ESM.doc]

##### Table S2. Results of the Pro-Pper/Pro-PNA^-^ cDNA-microarray hybridization analysis for positive and negative hybridization control spots. Pro-Pper/Pro-PNA^-^ fold changes (F) and standard deviations (SD) are detailed, as well as p-value, (α = 0.05). Absence of differential gene expression has been observed in positive controls (*p* ≥ 0.05). Mean fluorescence intensity (FI) and SD values are provided.

| ***Spot*** | | **F ± SD** | | ***p*** | | | | **Positive control** | |
| --- | --- | --- | --- | --- | --- | --- | --- | --- | --- |
| cLin79A1 | | 1.2 ± 0.2 | 0.271 | | | | *Li Polβ* | | |
| cLin79A2 | | 1.4 ± 0.1 | 0.103 | | | | *Li TopoII* | | |
| cLin79A3 | | 1.1 ± 0.2 | 0.329 | | | | *Li p36/LACK* | | |
| cLin79B1 | | -1.2 ± 0.2 | 0.072 | | | | *Li hsp70* | | |
| cLin79B2 | | 1.1 ± 0.2 | 0.114 | | | | *Ldo hsp70* | | |
| cLin79B3 | | 1.0 ± 0.0 | 0.253 | | | | *Lam hsp70* | | |
| cLin79C1 | | -1.2 ± 0.1 | 0.511 | | | | *Lma hsp70* | | |
| cLin79C2 | | 1.3 ± 0.5 | 0.447 | | | | *Li A2* | | |
| cLin79C3 | | 1.1 ± 0.1 | 0.136 | | | | *Ldo A2* | | |
| cLin79D1 | | 1.3 ± 0.2 | 0.152 | | | | *Li GAPDH* | | |
| cLin79D2 | | -1.1 ± 0.4 | 0.111 | | | | *Ldo GAPDH* | | |
| cLin79D3 | | 1.0 ± 0.2 | 0.409 | | | | *LigDNA* | | |
| cLin79H2 | | 1.0 ± 0.2 | 0.510 | | | | *Herring sperm DNA* | | |
| ***Spot*** | **Mean FI ± SD** | | | | **Negative control** | | | |  |
| cLin79E1 | | 25± 3 | | | | *Lfe nifA/hlyD* | | | |
| cLin79E2 | | 187 ± 110 | | | | *Lfe nifD/nifK* | | | |
| cLin79E3 | | 321 ± 34 | | | | *Lfe nifH* | | | |
| cLin79F1 | | 517 ± 47 | | | | *Lfe nifS/nifU* | | | |
| cLin79F2 | | 94 ± 13 | | | | *Lfe nifX/nifB* | | | |
| cLin79F3 | | 132 ± 32 | | | | *Lfe nifH/nifD* | | | |
| cLin79G1 | | 440 ± 243 | | | | *Lfe nifE* | | | |
| cLin79G2 | | 25 ± 12 | | | | *Lfe nifV/HesB* | | | |
| cLin79G3 | | 63 ± 53 | | | | *Lfe nifV* | | | |
| cLin79H1 | | 575 ± 218 | | | | *Lfe nifW/Bgene* | | | |
| cLin79H3 | | 20 ± 3 | | | | *1XSSC* | | | |
|  | |  | | | | | |  | |
