# Supplementary material for: In vitro infectivity and differential gene expression of Leishmania infantum metacyclic promastigotes: negative selection with peanut agglutinin in culture versus isolation from the stomodeal valve of Phlebotomus perniciosus
Source: BMC Genomics. 2016 May 20;17:375. doi: 10.1186/s12864-016-2672-8 (PMC4874012; doi:10.1186/s12864-016-2672-8)
Supplement: Additional file 3: — Hypothetical proteins. Table S3. Hypothetical protein genes up-regulated in Pro-Pper/Pro-PNA−. Table S4. Hypothetical protein genes down-regulated in Pro-Pper/Pro-PNA−. Table S5. Type c and qPCR non-determined clones. (DOC 255 kb) [file 12864_2016_2672_MOESM3_ESM.doc]

**Table S3. Hypothetical proteins up-regulated in Pro-Pper/Pro-PNA^-^.**

| ***Clone*** | ***F*** | ***Log_2_F ± SD*** | ***p*** | ***e-value*** | | ***Def.*** | ***Annotation*** | ***Annotated gene function*** | ***qRT-PCR*** | |
| --- | --- | --- | --- | --- | --- | --- | --- | --- | --- | --- |
|  |  |  |  | ***Fw*** | ***Rv*** |  |  |  |  |  |
| Lin99E6 | 8.00 | 3.0 ± 0.4 | 0.048 | 0 | 0 | b | LinJ.26.0710 | Hypothetical protein, conserved | N.D. |  |
| Lin100B4 | 2.90 | 1.5 ± 0.6 | 0.006 | 0 | 0 | b | LinJ.06.0050 | Hypothetical protein, conserved | N.D. |  |
| Lin100E10 | 2.00 | 1.0 ± 0.3 | 0.009 | 6 e-38 | 2 e-47 | a | LinJ.30.1690 | Hypothetical protein, conserved | N.D. |  |
| Lin103B5 | 4.36 | 2.1 ± 0.4 | 0.037 | 0 | 0 | a | LinJ.30.0810 | Proteína hipotética conservada | N.D. |  |
|  |  |  |  |  |  |  | LinJ.30.0820 | Proteína hipotética conservada | N.D. |  |
|  |  |  |  |  |  |  | LinJ.30.0830 | Oligo (U)-binding protein TBRGG1, putative | - | 1.5 ± 0.3 |
| Lin110G10 | 2.11 | 1.1 ± 0.2 | 0.034 | 1e-119 | 1e-112 | a | LinJ.30.1550 | Hypothetical protein, conserved | N.D. |  |
| Lin111C2 | 5.63 | 2.5 ± 0.9 | 0.023 | 3 e -38 | 1 e-29 | a | LinJ.30.1690 | Hypothetical protein, conserved | N.D. |  |
| Lin116G12 | 7.08 | 2.8 ± 0.7 | 0.043 | 0 | 0 | b | LinJ.01.0310 | Acidocalcisome exopolyphosphatase, putative | - | -1.2 ± 0.2 |
|  |  |  |  |  |  |  | LinJ.01.0320 | Hypothetical protein, conserved | N.D. |  |
|  |  |  |  |  |  |  | LinJ.01.0330 | Poly(A) export protein, putative | - | -1.4 ± 0.2 |
|  |  |  |  |  |  |  | LinJ.01.0340 | Hypothetical protein, conserved | N.D. |  |
|  |  |  |  |  |  |  | LinJ.01.0350 | Hypothetical protein, conserved | N.D. |  |
| Lin139D10 | 5.02 | 2.3 ± 0.5 | 0.020 | 0 | 0 | b | LinJ.30.1370 | Hypothetical protein, unknown function | N.D. |  |
| Lin139E9 | 4.0 | 2.0 ± 0.6 | 0.007 | 0 | 0 | b | LinJ.30.1370 | Hypothetical protein, conserved | N.D. |  |
| Lin140C10 | 3.30 | 1.7 ± 0.4 | 0.022 | 0 | 0 | b | LinJ.30.1370 | Hypothetical protein, unknown function | N.D. |  |
| Lin142C8 | 3.28 | 1.7 ± 0.4 | 0.025 | 0 | 0 | b | LinJ.33.1900 | Hypothetical protein, conserved | N.D. |  |
| Lin147B10 | 2.06 | 1.0 ± 0.4 | 0.032 | 0 | 0 | b | LinJ.31.1420 | Hypothetical protein, conserved | N.D. |  |
| Lin147C7 | 2.29 | 1.2 ± 0.4 | 0.042 | 0 | 0 | b | LinJ.08.0430 | Hypothetical protein, conserved | N.D. |  |
| Lin148B12 | 2.20 | 1.1 ± 0.2 | 0.007 | 0 | 0 | b | LinJ.09.1060 | Hypothetical protein, conserved | N.D. |  |
| Lin149E5 | 2.10 | 1.1 ± 0.5 | 0.008 | 0 | 0 | b | LinJ.30.2310 | Hypothetical protein, conserved | N.D. |  |
|  |  |  |  |  |  |  | LinJ.30.2320 | Hypothetical protein, conserved | N.D. |  |
|  |  |  |  |  |  |  | LinJ.30.2330 | Hypothetical protein, conserved | N.D. |  |
| Lin153C3 | 2.49 | 1.3 ± 0.5 | 0.015 | 0 | 0 | b | LinJ.31.0920 | Hypothetical protein, conserved | N.D. |  |
| Lin155C10 | 2.71 | 1.4 ± 0.2 | 0.041 | 0 | 0 | a | LinJ.22.1330 | Hypothetical protein, conserved | N.D. |  |
| Lin155H9 | 2.08 | 1.1 ± 0.7 | 0.008 | 0 | 0 | b | LinJ.28.1140 | Hypothetical protein, conserved | N.D. |  |
| Lin158A9 | 2.51 | 1.3 ± 0.4 | 0.039 | 0 | 0 | b | LinJ.30.2310 | Hypothetical protein, conserved | N.D. |  |
|  |  |  |  |  |  |  | LinJ.30.2320 | Hypothetical protein, conserved | N.D. |  |
|  |  |  |  |  |  |  | LinJ.30.2330 | Hypothetical protein, conserved | N.D. |  |
| Lin159A12 | 2.02 | 1.0 ± 0.4 | 0.040 | 0 | 0 | b | LinJ.30.2310 | Hypothetical protein, conserved | N.D. |  |
|  |  |  |  |  |  |  | LinJ.30.2320 | Hypothetical protein, conserved | N.D. |  |
|  |  |  |  |  |  |  | LinJ.30.2330 | Hypothetical protein, conserved | N.D. |  |
| Lin159D9 | 2.21 | 1.1 ± 0.6 | 0.035 | 0 | 0 | b | LinJ.35.2920 | Hypothetical protein, conserved | N.D. |  |
|  |  |  |  |  |  |  | LinJ.35.2930 | Hypothetical protein, conserved | N.D. |  |
| Lin159D10 | 2.76 | 1.5 ± 0.4 | 0.000 | 0 | 0 | b | LinJ.35.2920 | Hypothetical protein, conserved | N.D. |  |
| Lin170F11 | 2.00 | 1.0 ± 0.8 | 0.009 | 0 | 0 | b | LinJ.35.0940 | Hypothetical protein, conserved | N.D. |  |
| Lin171E5 | 2.67 | 1.4 ± 0.4 | 0.000 | 0 | 0 | a | LinJ.08.0040 | Hypothetical protein | N.D. |  |
|  |  |  |  |  |  |  | LinJ.08.0050 | Hypothetical protein, unknown function | N.D. |  |
|  |  |  |  |  |  |  | LinJ.08.0060 | Phosphoglycerate mutase, putative | - | 1.1 ± 0.0 |
|  |  |  |  |  |  |  | LinJ.08.0070 | Hypothetical protein, conserved | N.D. |  |
| Lin172B12 | 2.01 | 1.0 ± 0.2 | 0.009 | 0 | 0 | b | LinJ.31.1680 | Hypothetical protein, conserved | N.D. |  |
|  |  |  |  |  |  |  | LinJ.31.1690 | Hypothetical protein, conserved | N.D. |  |
|  |  |  |  |  |  |  | LinJ.31.1700 | Hypothetical protein, conserved | N.D. |  |
| Lin175B5 | 3.92 | 2.0 ± 0.3 | 0.010 | 0 | 0 | b | LinJ.03.0010 | Hypothetical protein | N.D. |  |
|  |  |  |  |  |  |  | LinJ.03.0020 | Hypothetical protein, conserved | N.D. |  |
|  |  |  |  |  |  |  | LinJ.03.0030 | D-3-phosphoglycerate dehydrogenase-like protein | - | -1.3 ± 0.2 |
| Lin179H9 | 2.45 | 1.3 ± 0.4 | 0.041 | 0 | 0 | b | LinJ.36.0580 | Hypothetical protein, conserved | N.D. |  |
|  |  |  |  |  |  |  | LinJ.36.0590 | Ubiquitin-like protein | - | 1.4 ± 0.3 |
| Lin181A10 | 4.00 | 2.0 ± 0.2 | 0.026 | 0 | 0 | b | LinJ.22.0610 | Hypothetical protein, conserved | N.D. |  |
| Lin181G1 | 2.20 | 1.1 ± 0.1 | 0.041 | 0 | 0 | b | LinJ.22.0050 | Hypothetical protein, conserved | N.D. |  |
| Lin181H2 | 3.53 | 1.8 ± 0.1 | 0.026 | 0 | 0 | a | LinJ.30.0830 | Oligo (U)-binding protein TBRGG1, putative | - | -1.5 ± 0.3 |
|  |  |  |  |  |  |  | LinJ.30.0840 | Hypothetical protein, conserved | N.D. |  |
| Lin182G11 | 4.92 | 2.3 ± 0.6 | 0.029 | 0 | 0 | b | LinJ.07.0020 | Hypothetical protein, conserved | N.D. |  |
|  |  |  |  |  |  |  | LinJ.07.0030 | Hypothetical protein, conserved | N.D. |  |
| Lin183B10 | 2.23 | 1.2 ± 0.5 | 0.019 | 0 | 0 | a | LinJ.06.1360 | Hypothetical protein, conserved | N.D. |  |
| Lin186B9 | 2.31 | 1.2 ± 0.4 | 0.002 | 0 | 0 | b | LinJ.30.2880 | Hypothetical protein, conserved | N.D. |  |
|  |  |  |  |  |  |  | LinJ.30.2890 | Hypothetical protein, conserved | N.D. |  |
| Lin194G11 | 2.60 | 1.4 ± 0.4 | 0.024 | 0 | 0 | a | LinJ.32.0010 | Hypothetical protein, conserved | N.D. |  |
|  |  |  |  |  |  |  | LinJ.32.0020 | Hypothetical protein, conserved | N.D. |  |
| Lin198E3 | 4.82 | 2.3 ± 0.4 | 0.006 | 0 | 9 e-179 | b | LinJ.06.1360 | Hypothetical protein, conserved | N.D. |  |
| Lin200B5 | 2.42 | 1.3 ± 0.4 | 0.017 | 0 | 0 |  | LinJ.32.3190 | Hypothetical protein, conserved | N.D. |  |
| Lin200D7 | 2.55 | 1.4 ± 0.8 | 0.025 | 0 | 0 | b | LinJ.36.0520 | Hypothetical protein, conserved | N.D. |  |
| Lin202E12 | 2.71 | 1.4 ± 0.1 | 0.009 | 0 | 0 | a | LinJ.07.0040 | Hypothetical protein, conserved | N.D. |  |
| Lin210B2 | 3.83 | 1.9 ± 0.3 | 0.024 | 0 | 0 | b | LinJ.31.2340 | Hypothetical protein, conserved | N.D. |  |
|  |  |  |  |  |  |  | LinJ.31.2350 | ADP-ribosylation factor | - | -1.2 ± 0.0 |
| Lin210D4 | 6.24 | 2.6 ± 0.2 | 0.020 | 0 | 7e-167 | b | LinJ.22.0620 | Hypothetical protein, conserved | N.D. |  |
| Lin211E4 | 2.83 | 1.5 ± 0.1 | 0.006 | 0 | 0 | b | LinJ.31.0960 | Hypothetical protein, conserved | N.D. |  |
|  |  |  |  |  |  |  | LinJ.31.0970 | Hypothetical protein, conserved | N.D. |  |
|  |  |  |  |  |  |  | LinJ.31.0980 | Hypothetical protein, conserved | N.D. |  |
| Lin219A3 | 2.14 | 1.1 ± 0.1 | 0.012 | 0 | 0 | b | LinJ.31.2340 | Hypothetical protein, conserved | N.D. |  |
| Lin221A4 | 2.16 | 1.1 ± 0.2 | 0.016 | 0 | 0 | b | LinJ.36.1500 | Hypothetical protein, conserved | N.D. |  |
| Lin227B12 | 3.69 | 1.9 ± 0.1 | 0.007 | 2e-173 | 5e-74 | a | LinJ.31.1000 | Hypothetical protein, conserved | N.D. |  |
| Lin228C4 | 2.54 | 1.3 ± 0.2 | 0.041 | 0 | 0 | b | LinJ.31.2310 | Hypothetical protein, unknown function | N.D. |  |
| Lin228D4 | 6.45 | 2.7 ± 0.1 | 0.038 | 0 | 0 | a | LinJ.19.0080 | Hypothetical protein, conserved | N.D. |  |
|  |  |  |  |  |  |  | LinJ.19.0090 | Fibrillarin, putative | - | -1.1 ± 0.5 |
| Lin230E9 | 2.32 | 1.2 ± 0.4 | 0.020 | 0 | 0 | b | LinJ.36.6490 | ADP-ribosylation factor, putative | - | 1.2 ± 0.2 |
|  |  |  |  |  |  |  | LinJ.36.6500 | Hypothetical protein, conserved | N.D. |  |
| Lin238F5 | 6.81 | 2.8 ± 0.2 | 0.045 | 0 | 0 | a | LinJ.03.0020 | Hypothetical protein, conserved | N.D. |  |
| Lin240G12 | 2.33 | 1.2 ± 0.1 | 0.018 | 0 | 0 | b | LinJ.22.0620 | Hypothetical protein, conserved | N.D. |  |
| Lin241D5 | 2.21 | 1.1 ± 0.3 | 0.021 | 0 | 0 | a | LinJ.17.0020 | Hypothetical protein, conserved | N.D. |  |
|  |  |  |  |  |  |  | LinJ.17.0030 | Hypothetical protein, conserved | N.D. |  |
| Lin250G5 | 2.87 | 1.5 ± 0.3 | 0.035 | 0 | 0 | b | LinJ.09.1610 | Hypothetical protein, conserved | N.D. |  |
| Lin252G1 | 2.09 | 1.1 ± 0.1 | 0.006 | 0 | 0 | a | LinJ.34.4040 | Hypothetical protein, conserved | N.D. |  |
| Lin270A1 | 3.08 | 1.6 ± 0.5 | 0.018 | 0 | 0 | b | LinJ.11.0980 | Hypothetical protein, conserved | N.D. |  |
|  |  |  |  |  |  |  | LinJ.11.0990 | Adaptin-like protein | - | -1.1 ± 0.2 |
| Lin280H4 | 2.71 | 1.4 ± 0.5 | 0.019 | 0 | 0 | b | LinJ.34.0780 | Hypothetical protein, conserved | N.D. |  |
|  |  |  |  |  |  |  | LinJ.34.0790 | Hypothetical protein, conserved | N.D. |  |
| Lin299D9 | 2.43 | 1.3 ± 0.3 | 0.030 | 0 | 0 | b | LinJ.17.0970 | Hypothetical protein, conserved | N.D. |  |
| Lin311E4 | 2.15 | 1.1 ± 0.2 | 0.034 | 0 | 2e-121 | a | LinJ.31.0760 | Hypothetical protein, conserved | N.D. |  |
|  |  |  |  |  |  |  | LinJ.31.0770 | Hypothetical protein, conserved | N.D. |  |

**Table S4. Hypothetical proteins down-regulated in Pro-Pper/Pro-PNA^-^.**

| ***Clone*** | ***F*** | ***Log_2_F ± SD*** | ***p*** | ***e-value*** | | ***Def.*** | ***Annotation*** | ***Annotated gene function*** | ***qRT-PCR*** | |
| --- | --- | --- | --- | --- | --- | --- | --- | --- | --- | --- |
|  |  |  |  | ***Fw*** | ***Rv*** |  |  |  |  |  |
| Lin43D8 | -2.11 | -1.1 ± 0.4 | 0.017 | 0 | 0 | a | LinJ.13.0520 | Hypothetical protein, unknown function | N.D. |  |
|  |  |  |  |  |  |  | LinJ.13.0530 | Hypothetical protein, conserved | N.D. |  |
| Lin100E12 | -2.66 | -1.4 ± 0.2 | 0.049 | 0 | 0 | a | LinJ.23.0730 | Hypothetical protein, conserved | N.D. |  |
| Lin105F3 | -2.46 | -1.3 ± 0.1 | 0.019 | 0 | 0 | a | LinJ.30.2400 | Hypothetical protein, conserved | N.D. |  |
| Lin106H2 | -3.17 | -1.7 ± 0.2 | 0.002 | 6e-161 | 0 | b | LinJ.33.0840 | Hypothetical protein, conserved | N.D. |  |
| Lin107F2 | -2.07 | -1.0 ± 0.1 | 0.020 | 0 | 0 | b | LinJ.09.0430 | Hypothetical protein, conserved | N.D. |  |
| Lin109G10 | -2.02 | -1.0 ± 0.3 | 0.042 | 0 | 0 | a | LinJ.26.2040 | Hypothetical protein, conserved | N.D. |  |
| Lin111H4 | -2.05 | -1.0 ± 0.4 | 0.027 | 1e-66 | 3e-73 | a | LinJ.18.0240 | Hypothetical protein, conserved | N.D. |  |
| Lin121E9 | -2.25 | -1.2 ± 0.3 | 0.004 | 0 | 0 | a | LinJ.36.4430 | Hypothetical protein, conserved | N.D. |  |
|  |  |  |  |  |  |  | LinJ.36.4440 | Hypothetical protein, conserved | N.D. |  |
| Lin123G10 | -2.44 | -1.3 ± 0.2 | 0.040 | 0 | 1e-119 | b | LinJ.31.0560 | Hypothetical protein, conserved | N.D. |  |
|  |  |  |  |  |  |  | LinJ.31.0570 | Hypothetical protein, conserved | N.D. |  |
| Lin131H9 | -2.03 | -1.0 ± 0.3 | 0.011 | 0 | 0 | b | LinJ.04.0740 | Hypothetical protein, conserved | N.D. |  |
| Lin135D9 | -2.33 | -1.2 ± 0.2 | 0.017 | 0 | 0 | b | LinJ.15.0060 | Hypothetical protein, conserved | N.D. |  |
|  |  |  |  |  |  |  | LinJ.15.0070 | Hypothetical protein, conserved | N.D. |  |
| Lin139G2 | -4.42 | -2.1 ± 0.3 | 0.028 | 0 | 0 | b | LinJ.35.1710 | Hypothetical protein, conserved | N.D. |  |
| Lin153H12 | -2.78 | -1.5 ± 0.1 | 0.018 | 2e-43 | 3e-52 | b | LinJ.30.2310 | Hypothetical protein, conserved | N.D. |  |
|  |  |  |  |  |  |  | LinJ.30.2320 | Hypothetical protein, conserved | N.D. |  |
|  |  |  |  |  |  |  | LinJ.30.2330 | Hypothetical protein, conserved | N.D. |  |
| Lin162B4 | -2.09 | -1.1 ± 0.2 | 0.026 | 0 | 0 | a | LinJ.14.0380 | Hypothetical protein, conserved | N.D. |  |
| Lin170D5 | -2.00 | -1.0 ± 0.3 | 0.005 | 0 | 0 | a | LinJ.22.0620 | Hypothetical protein, conserved | N.D. |  |
| Lin170F3 | -2.03 | -1.0 ± 0.5 | 0.018 | 0 | 0 | b | LinJ.31.2220 | Hypothetical protein, unknown function | N.D. |  |
| Lin170G3 | -2.38 | -1.2 ± 0.1 | 0.019 | 0 | 0 | b | LinJ.03.0800 | Hypothetical protein, conserved | N.D. |  |
| Lin172C3 | -2.23 | -1.1 ± 0.2 | 0.007 | 0 | 1e-173 | a | LinJ.12.0210 | Hypothetical protein, conserved | N.D. |  |
| Lin194D5 | -2.33 | -1.2 ± 0.3 | 0.013 | 0 | 0 | a | LinJ.30.0810 | Hypothetical protein, conserved | N.D. |  |
| Lin197E6 | -3.88 | -2.0 ± 0.0 | 0.040 | 0 | 0 | a | LinJ.30.2400 | Hypothetical protein, conserved | N.D. |  |
| Lin198F7 | -2.65 | -1.4 ± 0.5 | 0.009 | 0 | 0 | a | LinJ.34.2220 | Hypothetical protein, conserved | N.D. |  |
| Lin201A3 | -2.01 | -1.0 ± 0.1 | 0.016 | 0 | 0 | a | LinJ.09.1050 | Hypothetical protein, conserved | N.D. |  |
| Lin201A11 | -3.74 | -1.9 ± 0.2 | 0.000 | 0 | 0 | b | LinJ.09.0280 | Hypothetical protein, conserved | N.D. |  |
| Lin201E2 | -2.32 | -1.2 ± 0.2 | 0.003 | 0 | 0 | b | LinJ.15.0930 | Hypothetical protein, conserved | N.D. |  |
|  |  |  |  |  |  |  | LinJ.15.0940 | Myo-inositol 1-phosphatase, putative | - | 1.1 ± 0.0 |
| Lin202G9 | -2.13 | -1.1 ± 0.1 | 0.011 | 0 | 0 | a | LinJ.30.1760 | Hypothetical protein, conserved | N.D. |  |
| Lin203H5 | -2.08 | -1.0 ± 0.2 | 0.011 | 0 | 0 | a | LinJ.23.0800 | Hypothetical protein, conserved | N.D. |  |
| Lin205D8 | -2.26 | -1.2 ± 0.1 | 0.045 | 0 | 0 | b | LinJ.09.0810 | Hypothetical protein, conserved | N.D. |  |
| Lin208D11 | -2.09 | -1.1 ± 0.2 | 0.010 | 0 | 0 | b | LinJ.09.0550 | Hypothetical protein, conserved | N.D. |  |
| Lin221E10 | -2.23 | -1.2 ± 0.2 | 0.010 | 0 | 0 | a | LinJ.22.0920 | Hypothetical protein, conserved | N.D. |  |
| Lin223F2 | -5.10 | -2.3 ± 1.3 | 0.014 | 0 | 0 | b | LinJ.13.1460 | - | N.D. |  |
| Lin230D7 | -2.34 | -1.2 ± 0.4 | 0.002 | 0 | 0 | a | LinJ.06.0200 | Hypothetical protein, conserved | N.D. |  |

**Table S5. Type c and qPCR non-determined clones.**

| *Clone* | *F* | *Log_2_F ± SD* | *p* | *e-value* | | *Def.* | *Id.* | *Annotated gene function* |
| --- | --- | --- | --- | --- | --- | --- | --- | --- |
|  |  |  |  | *Fw* | *Rv* |  |  |  |
| Lin200B5 | 2.42 | 1.3 ± 0.4 | 0.017 | 5e-177 | 0 | c | LinJ.31.3190 | Hypothetical protein, conserved |
| Lin233E10 | 2.25 | 1.2 ± 0.4 | 0.039 | 0 | 0 | c | LinJ.34.2170 | Hypothetical protein, conserved |
|  |  |  |  |  |  |  | LinJ.15.1300 | Hypothetical protein, conserved |
| Lin122H5 | -2.10 | -1.1 ± 0.0 | 0.023 | 0 | 0 | c | LinJ.10.0710 | Hypothetical protein, unknown function |
|  |  |  |  |  |  |  | LinJ.32.1080 | Hypothetical protein, conserved |
| Lin140C6 | -2.02 | -1.0 ± 0.3 | 0.012 | - | 0 | c | LinJ.31.1870 | Hypothetical protein, conserved |
| Lin181E2 | -2.63 | -1.4 ± 0.4 | 0.009 | 0 | 0 | c | LinJ.22.1480 | Hypothetical protein, conserved |
|  |  |  |  |  |  |  | LinJ.36.6840 | Hypothetical protein, conserved |
| Lin199D4 | -2.60 | -1.4 ± 0.0 | 0.019 | 0 | - | c | LinJ.35.0730 | Hypothetical protein, conserved |
| Lin223B12 | -3.49 | -1.8 ± 0.6 | 0.020 | 0 | 0 | c | LinJ.15.0380 | Hypothetical protein, conserved |
| Lin246E10 | -2.38 | -1.2 ± 0.3 | 0.024 | - | 0 | c | LinJ.15.0490 | Hypothetical protein |
